# Use of Okadaic Acid to Identify Relevant Phosphoepitopes in Pathology: A Focus on Neurodegeneration

**DOI:** 10.3390/md11051656

**Published:** 2013-05-21

**Authors:** Miguel Medina, Jesús Avila, Nieves Villanueva

**Affiliations:** 1 CIBERNED (Center for Networked Biomedical Research in Neurodegenerative Diseases), Valderrebollo 5, Madrid 28041, Spain; E-Mail: mmedina@ciberned.es; 2 Center of Molecular Biology “Severo Ochoa” CSIC-UAM, Nicolás Cabrera 1, Madrid 28049, Spain; 3 National Center of Microbiology (CNM), Carlos III Institute of Health (ISCIII), Crta. Majadahonda-Pozuelo km 2, Majadahonda, Madrid 28220, Spain; E-Mail: nvilla@isciii.es

**Keywords:** Alzheimer, GSK-3, kinases, neurodegeneration, okadaic acid, phosphatases, phosphorylation, PP2A, tangles, tau

## Abstract

Protein phosphorylation is involved in the regulation of a wide variety of physiological processes and is the result of a balance between protein kinase and phosphatase activities. Biologically active marine derived compounds have been shown to represent an interesting source of novel compounds that could modify that balance. Among them, the marine toxin and tumor promoter, okadaic acid (OA), has been shown as an inhibitor of two of the main cytosolic, broad-specificity protein phosphatases, PP1 and PP2A, thus providing an excellent cell-permeable probe for examining the role of protein phosphorylation, and PP1 and PP2A in particular, in any physiological or pathological process. In the present work, we review the use of okadaic acid to identify specific phosphoepitopes mainly in proteins relevant for neurodegeneration. We will specifically highlight those cases of highly dynamic phosphorylation-dephosphorylation events and the ability of OA to block the high turnover phosphorylation, thus allowing the detection of modified residues that could be otherwise difficult to identify. Finally, its effect on tau hyperhosphorylation and its relevance in neurodegenerative pathologies such as Alzheimer’s disease and related dementia will be discussed.

## 1. Introduction

Okadaic acid is a lipophilic natural compound originally isolated from the marine black sponges *Halichondria okadaii* and *Halichondria melanodocia* [1] and it was subsequently shown to be produced by several marine dinoflagellates belonging to the genera *Dinophysis* and *Prorocentrum* [2,3]. This polyether fatty acid compound is almost exclusively accumulated in the mussel digestive gland and consumption of these mollusks might lead to a syndrome in humans known as diarrheic shellfish poisoning (DSP) due to the toxic effects of OA and its analogs, collectively known as dinophysistoxins [4,5,6].

Due to the severe gastrointestinal symptoms associated to DSP, OA is primarily considered to be an enterotoxin [7,8], causing epithelial damage and fluid accumulation in the gastrointestinal tract that eventually, at high doses, may lead to death. However, low oral doses have also been shown to lead to systemic immunotoxicity in mice [9] and *in vivo* studies in mice, have reported OA distribution and excretion after oral administration as well as morpho-functional changes in several organs targeted by the toxin [10]. Thus, the target organ and the severity of OA-induced toxic effects depend on the dose and the route of administration [11]. For instance, when administered intravenously, OA is highly hepatotoxic with undetectable effects on the intestine but also has an impact on cytoskeletal elements at sub-lethal doses [12]; or when applied to mouse skin OA causes severe irritation. In addition, *Halichondria okadaii* crude extracts had already been shown in the 1970s to show remarkable cytotoxic activity. Several polyethers, including OA and halichondrins, were later isolated and shown to be responsible for the anti-mitotic activity associated with the crude natural extracts [13]. Finally, 25 years after the first report of tumor promotion by OA in [14], it is now well established that the toxin is functionally a potent tumor promoter in various organs, including skin, stomach and liver [15,16].

## 2. Molecular and Cellular Effects

OA was first identified as a potent phosphatase inhibitor about a quarter of century ago in a pioneer study [17] and its toxicity has since been generally attributed to its effect on cellular phosphatases, although some authors have recently put a causal effect into question [18]. OA is a potent inhibitor of two major Ser/Thr protein phosphatases present in mammalian cells, PP2A and PP1, with IC_50_ values of 0.2 nM and 20 nM, respectively, but potently inhibits other phosphatases as well, such as PP4 and PP5 [19,20]. In addition OA it is also able to inhibit, albeit to a much lesser extent (about 100-fold selectivity), other phosphatases such as PP2B or PP7 while having virtually no effect on Ser/Thr phosphatases from the PPM (protein phosphatase Mg^2+^- or Mn^2+^-dependent) family or the tyrosine phosphatases [20]. 

OA is the archetypal member of an entire class of remarkably distinct secondary metabolites from such disparate organisms as bacteria, blue-green algae, dinoflagellates, red algae, and even insects, that together comprise the “okadaic acid class of phosphatase inhibitors” [13]. Thus, the marine natural product OA is perhaps the most well-known member of a diverse array of secondary metabolites that have emerged as valuable probes for studying the roles of various cellular protein Ser/Thr phosphatases and has become one of the world’s most widely used marine natural product in biological research.

Total synthesis of OA has been achieved and has allowed detailed topological and intermolecular recognition studies, largely through X-ray crystallography [13]. This information has then sparked considerable structure activity relationship studies directed toward the development of phosphatase-based therapeutics compounds [21] and has opened the door for the use of small molecule inhibitors to study the roles of sensitive protein phosphatases.

Apart from being quite a stable compound, OA is a fairly hydrophobic compound that can readily enter living cells, being able to cross the plasma membrane and block the dephosphorylation of proteins that are substrates for several protein kinases and constitutes, therefore, an excellent tool to analyze biological properties that are regulated through reversible protein phosphorylation [22,23].

The lack of sufficient specificity leads to relatively indiscriminant phosphatase inhibition though, and may simultaneously affect a variety of important cellular processes in addition to the targeted ones. Hence, we must be cautious to assign cellular functions to specific phosphatases based only on cellular effects observed after treatment with OA or its derivatives. Indeed to date, although a more selective inhibitor has been identified (fostriecin), OA remains the most widely used inhibitor in studies designed to provide insight into the biological actions of PP1 and PP2A.

Thus, under normal *in vitro* assay conditions, PP2A is completely inhibited by 1 nM OA, while PP1 is unaffected at this concentration; however the use of this inhibitor to discriminate between the effects of the two target enzymes is not as powerful in living cells as it is on recombinant enzymes or in cell extracts. Cellular inhibition constants may be influenced by a number of factors, including intracellular concentrations of either competing target enzymes or effectors that may compete at the inhibitor binding site, cell permeability, half-life of compounds, *etc.* [24]. Since intracellular concentrations of PP2A and PP1 may vary between the different cell types, often lying within the range of 0.1–1.0 μM [22], the right dose and time of treatment must be determined for each specific cell line [24,25].

Among the substrates already identified in different cell types, mounting evidence suggest that the cytoskeleton, a structure finely integrated to many cell functions, is particularly vulnerable to the toxic mechanisms induced by OA and related compounds, as altered phosphatase activity induced by pharmacological treatment with OA is accompanied by decreased cell adhesion and cytoskeletal reorganization [11,26]. On the other hand, it is noteworthy that OA and its derivatives do not bind phorbol ester receptors in cell membranes or activate protein kinase C *in vitro*. This lack of interaction with the phorbol ester receptor has led to the development of a new mechanistic pathway for tumor promotion [27,28].

Although conventional wisdom in the past 20 years has maintained that phosphatase inhibition is not only responsible for the intestinal effects of OA and derivatives, but also for their acute toxic effects, their tumor promoting activity and their neuronal toxicity, this mechanism of action has recently been questioned [18]. Despite this, OA’s protein phosphatases’ inhibition ability has been utilized in the development of sensitive assays for DSPs in shellfish [29,30]. 

## 3. Using OA to Identify Highly Dynamic Phosphorylation Sites

Phosphorylation, the reversible addition of a phosphate group to amino acid side chains of proteins, is a fundamental regulator of protein activity, stability, and molecular interactions. Most cellular processes such as inter- and intracellular signaling, protein synthesis, degradation, and apoptosis, rely on phosphorylation. This post-translational modification is thus involved in many diseases, rendering localization and assessment of extent of phosphorylation of major scientific interest. Therefore, the discovery and characterization of phosphoproteins, and the ability to identify specific phosphorylation sites, is of paramount importance for studying the molecular pathways that are regulated by reversible phosphorylation of proteins relevant in many cellular processes such as signal transduction, cell division and memory, among many others. Within this context, OA has emerged as an excellent tool for identifying and studying the myriad of events associated with the inhibition of protein Ser/Thr phosphatases and the reversible phosphorylation of proteins [22,23]. 

The ability of OA to inhibit PP2A/PP1 phosphatases has been used to facilitate the analysis of phosphorylated residues in many processes. Mainly, in cases of highly dynamic phosphorylation-dephosphorylation, OA treatment stops the high turnover phosphorylation allowing the detection of modified residues that could be difficult for identification under other circumstances. One case in point is that of some viral proteins, such as the human respiratory syncytial virus (HRSV) P protein [31]. This phosphorylated structural protein presents two types of phosphorylation sites, with phosphates added to a group of residues, a slower turnover is observed which can be detected in the absence of protein phosphatase inhibition. Whereas, inhibition of PP2A/PP1 with OA, increases the level of phosphorylation at some specific phosphosites, suggesting a more rapid turnover [32]. Actually, viral infection may also allow the high-turnover phosphorylation of proteins associated to the P proteins and the viral RNA polymerase complex [33,34]. Thus, OA treatment can be used to determine which amino acid residues incorporate high turnover phosphates during viral infection or other cellular processes. OA has in fact been shown to allow identification of cryptic phosphorylation sites *in vivo* that were not apparent in the absence of the inhibitor [35].

## 4. OA-Induced Neurodegeneration

OA was identified as a potent neurotoxin for cultured neuronal cells quite early on [36], as it is able to induce rapid time- and dose-dependent apoptotic changes in those cells [37]. Thus, different cell lines and primary neuronal cultures have been used to establish cellular models of OA-induced neurodegeneration, including human neuroblastoma cell lines [38,39], primary neuronal cultures [40,41,42] or even organotypic 3D cultures [43].

Not surprisingly, treatment of cultured cells with OA affects many different cellular pathways and numerous studies have been performed to clarify the various potential mechanisms of action or to look for ways to protect against OA-induced neurotoxicity. Thus, for instance, some authors have suggested that treatment of human neuroblastoma SH-SY5Y cells with OA may increase tau phosphorylation (see below) through sustained activation of the l-voltage-sensitive calcium channel [44] while others point to a role of GSK-3 in regulating tau phosphorylation and total tau levels [40]. On the other hand, different authors have involved calpain activation in OA toxicity in primary cortical neurons [45] and shown that pharmacological calpain inactivation can protect neurons against OA-induced neurodegeneration [46]. Another recent study worth mentioning shows that both α7 and β2* nicotinic ACh receptors can afford neuroprotection against OA neurotoxicity in human neuroblastoma cells, independently of Ca^++^ but involving the PI3K/Akt pathway [47]. Lastly, use of OA in cortical neurons has recently led to the identification of a novel signaling role of PP2A through the prolyl-isomerase Pin1 [48] which was proposed as a therapeutic target to reduce aberrant phosphorylation of neurofilament proteins in neurodegenerative disorders such as Alzheimer’s disease (AD), Parkinson’s disease, and amyotrophic lateral sclerosis.

Moreover, a recent work has used suppression subtractive hybridization in SH-SY5Y cells to identify genes that are differentially expressed after OA exposure [49]. A total of 247 subtracted clones (114 genes were up-regulated and 133 were down-regulated) which shared high homology with known genes were isolated, although only a handful were validated by quantitative real-time PCR. Most of these genes are involved in relevant cell functions such as metabolism, transport, translation, signal transduction and cell cycle, stressing the highly pleiotropic consequences of OA treatment.

Organotypic cultures made from slices of developing brain tissue preserve complex multi-cellular circumstances to a considerable extent—though not perfectly—and hence they have also been used to establish OA-induced neurodegeneration models. For instance, 400 μm-thick rat brain slices can be kept under metabolically active conditions in oxygenated (95% O_2_, 5% CO_2_) artificial cerebrospinal fluid (CSF) and treated with 1.0 μM OA for 1 h. Under these conditions, PP2A activity was shown to decrease up to a third of the vehicle-treated control slices, while activities of PP1 and PP2B were not affected [50]. A dramatic increase in the phosphorylation/activation of ERK1/2, MEK1/2, and p70 S6 kinase was observed in the OA-treated slices both by immunohistochemically and by Western blots using phosphorylation-dependent antibodies against these kinases. Furthermore, treatment of 6 μm sections of the OA-treated slices with purified PP2A reversed the phosphorylation/activation of these kinases. 

In addition, OA also shows neurotoxicity *in vivo*. In an attempt to produce an animal model of AD-like neurofibrillary degeneration, OA was injected into the cerebral cortex of adult sheep [51], leading to the appearance of Alz50 immunoreactive dystrophic neurites. Later on, OA injection in rat hippocampus was used to assess its neurotoxicity *in vivo* [52,53], and shown to induce dose-dependent damage, including neuronal death, loss of MAP2 immunostaining and increased expression of heat shock proteins. Numerous studies have since then shown that when injected into the brains of rodents, OA induces neuronal damage and neuropathological changes reminiscent of those seen in Alzheimer’s disease, including cognitive deficits such as memory impairment, as well as increased astrogliosis, oxidative stress, and neuronal death [54,55,56,57]. Since PP2A activity has been shown to be decreased in the brains of patients with AD [58], *in vivo* use of OA has also been revealed as an excellent way to understand diseases accompanied by protein hyperphosphorylation and cognitive deficits [38], as it is believed that protein hyperphosphorylation is due to inhibition of phosphatases *in vivo* and induces neuronal stress and subsequent neurodegeneration.

As noted below, intracerebral injection of OA causes tau hyperphosphorylation, formation of neurofibrillary tangles and deposits of β-amyloid, together with memory loss and neurodegeneration. It has been therefore suggested that intracerebral injection of OA, through its ability to inhibit protein phosphatases, would provide a useful model of Alzheimer’s disease [59]. 

## 5. Effects of OA on Tau Phosphorylation

As already mentioned, changes in the brain activity of protein phosphatases have been implicated in the pathogenesis of neurodegenerative diseases such as Alzheimer’s disease (AD) [60]. AD is a neurodegenerative disorder characterized by progressive memory loss and cognitive impairment while constituting the most common form of dementia among the elderly. The brains of patients suffering AD present the two classical hallmark histopathological lesions already described by Alois Alzheimer over a century ago, the extracellular “senile plaques” consisting of β-amyloid (Aβ) peptide, and intracellular neurofibrillary “tangles” (NFT) made up of hyperphosphorylated tau protein [61]. 

Tau protein is a microtubule-associated protein that in normal physiological conditions binds to and regulates assembly, dynamic behavior, and spatial organization of microtubules [62,63]. Within neurons, tau is predominantly found in axons as a highly soluble phosphoprotein where it also regulates the axonal transport of organelles, including mitochondria [64]. Tau is primarily, though not exclusively, a neuronal protein encoded by a single gene but with six major isoforms derived by alternative splicing [65,66]. Upon abnormal phosphorylation, the microtubule-associated protein tau reduces its affinity for and dissociates from microtubules. In AD brains, tau accumulates in the neuronal perikarya and processes as paired helical filaments (PHF) [67]. It has been suggested that at the single-cell level the defects start with a modification of tau by phosphorylation, resulting in a destabilization of microtubules giving rise to a “pre-tangle” stage. After this stage, the destabilization of microtubules leads to loss of dendritic microtubules and synapses, plasma membrane degeneration, and eventually cell death [68]. Furthermore, soluble abnormally phosphorylated tau can sequester both normal tau and HMW-MAPs and disassemble microtubules which most likely will also have implications for microtubule function [69,70].

In addition to AD, a number of other neurodegenerative disease present prominent tau pathology in the Central Nervous System (CNS), predominantly within the neuronal compartment, but also within glial cells. Because of this shared histopathological feature, they are referred collectively as tauopathies [71]. In tauopathies, the intracellular soluble tau forms abnormal, fibrillar structures of aggregated, hyperphosphorylated, and ubiquinated tau, which are associated with synaptic and neuronal loss. The occurrence of fibrillar tau inclusions in tauopathies strongly supports a key role in the observed clinical symptoms and pathology. 

Phospho-tau may be toxic inside neurons of the dentate gyrus [72], although inherent toxicity of phosphorylated tau has been a matter of debate [73]. Once neuronal degeneration takes place, intracellular tau is released to the extracellular space and can be found in the cerebrospinal fluid [74], but the possibility of tau being released by exocytosis has also received some attention recently [41,75]. There are up to 80 potential phosphorylation sites in the largest tau molecule, most of them located at the proline-rich and *C*-terminal regions [76] and around 40 of them have been shown to be actually phosphorylated in PHF-tau [77,78]. However, the relative importance of specific individual phosphorylation sites or groups for tau function and/or pathogenesis remains to be firmly established. In tauopathies, including AD, tau abnormalities—whether they are due to mutations in the tau gene or an altered 4R:3R tau ratio—cause brain deposition of highly phosphorylated tau in an aberrant conformation [79].

Hyperphosphorylation of tau has been suggested to be caused by an imbalance between kinase and phosphatase activities within the neurons during the development of Alzheimer’s disease [60,80,81]. We and others have shown many years ago that OA can be used to increase tau phosphorylation in cultured cells [82,83] and have used it, in combination with kinase inhibitors, as a tool to identify protein kinases responsible for phosphorylating specific residues [24]. 

Earlier studies have shown that a similar pattern of tau hyperphosphorylation than that observed in AD brains, including AD-related phosphoepitopes recognized by specific antibodies, can be obtained in cultured cells after OA treatment [82] and also *in vivo* after OA injection or microinfusion in rat hippocampus [38]. This increase in tau phosphorylation was believed to be a consequence of inhibiting PP2A since this enzyme is predominantly responsible for the dephosphorylation of this protein [84].

Hyperphosphorylation of tau at several abnormal hyperphosphorylation sites, as seen in AD brain, can be observed in OA-treated brain slices [50], as it has been suggested that the decrease in PP2A activity in AD brain may cause the activation of ERK1/2, MEK1/2, and p70 S6 kinase, and the abnormal hyperphosphorylation of tau, both via increased phosphorylation and decreased dephosphorylation. Organotypic cultures of brain slices have also been used to explore the effect of acute energy crisis on tau phosphorylation and the underlying mechanisms [85]. Tau was, unexpectedly, significantly dephosphorylated at different phosphoepitopes by acute anoxia for 30 min or 120 min whereas the activity of PP2A and the level of dephosphorylated PP2A catalytic subunit at residue Tyr307 were simultaneously increased and the active forms of ERK1/2 and JNK1/2 were decreased under anoxic incubation. Treatment of slices with 0.75 μM OA completely prevented tau from acute anoxia-induced dephosphorylation and restored the active forms of ERK1/2 and JNK1/2 to the control level. Finally, some studies have shown inhibition of PP1 and PP2A after intracerebral injection of OA into rat brains [53].

As already discussed, phosphorylation at specific sites can affect tau physiological functions, including its role in binding to and stabilizing the neuronal cytoskeleton. Tau aberrant phosphorylation could render it susceptible to potentially pathogenic alterations, including conformational changes, proteolytic cleavage and aggregation. While strategies that reduce tau phosphorylation in transgenic models of disease have been promising, our understanding of the mechanisms through which tau becomes abnormally phosphorylated in disease is lacking and thus OA will surely remain as having a key role as a tool to gain further insight into tau phosphorylation events critical to the process of neurodegeneration. As a sample, OA has been used by the pharma industry to set up cell-based primary screening assays to look for inhibitors of tau phosphorylation at specific sites such as Ser422 [86]. Another study used human neuroblastoma SH-SY5Y cells to establish a novel cell-based three-dimensional tauopathy model that showed advanced characteristics of matured neurons in comparison to monolayer cultures, without the need of artificial differentiation promoting agents and in which the neurodegenerative effects could be analyzed in real time with high sensitivity using a microcavity array-based impedance spectroscopy measurement system [87]. This model uses OA to induce tau hyperphosphorylation in specific AD sites and neurodegeneration and has been proposed as a high content analysis-based drug screening assay. 

Interestingly, OA neurotoxicity features have been recently used to set up a novel experimental model for Alzheimer studies in which a gradient of OA is generated over a neurite network that has been created in a microfluidic device connecting two separated cell compartments [88]. This further underlines the usefulness of OA as a wonderful biochemical tool for *in vitro* cell-based and *in vivo* studies involving protein phosphorylation events. 

## 6. Conclusions

As the prototypic and first recognized member of the “okadaic acid class” of phosphatase inhibitors, the marine natural product, OA, is arguably the most well-known member of a diverse array of secondary metabolites that have emerged as valuable probes for studying the roles of various cellular protein Ser/Thr phosphatases. We have briefly provided a historical perspective on the role that OA has played in stimulating a broad spectrum of modern, cutting-edge scientific research, as a consequence of its ability to inhibit an important group of protein Ser/Thr phosphatases. OA treatment stops high turnover phosphorylation events and allows the detection of phospho-epitope that may be difficult to identify under other circumstances. OA is already being employed in basic studies directed towards understanding such diverse human disease-related processes such as cancer, AIDS and other viral infections, inflammation, osteoporosis, Alzheimer’s disease, and diabetes. OA has also been widely used to establish a variety of cellular and animal models of OA-induced neurodegeneration as treatment of cells or injection of the compound into the brain of rodents brings about several AD-like pathological characteristics. Phosphorylation at specific sites can influence the physiological functions of tau protein. While strategies that reduce tau phosphorylation in transgenic models of disease are promising, our understanding of the mechanisms through which tau becomes abnormally phosphorylated in disease is lacking. Thus, OA definitely remains a powerful tool in future studies aimed at identifying relevant phosphoepitopes and clarifying their role in many physiological and pathological processes.
